# A systematic review of palliative care tools and interventions for people with severe mental illness

**DOI:** 10.1186/s12888-019-2078-7

**Published:** 2019-04-03

**Authors:** Karin den Boer, Anke J. E. de Veer, Linda J. Schoonmade, Kim J. Verhaegh, Berno van Meijel, Anneke L. Francke

**Affiliations:** 1 Nivel. Netherlands Institute for Health Services Research, P.O. Box 1568, Utrecht, 3500 BN The Netherlands; 2 Medical Library, VU University Amsterdam, Amsterdam, the Netherlands; 3 Amsterdam UMC, VU University Medical Center, Department of Psychiatry, Amsterdam Public Health Research Institute, Amsterdam, the Netherlands; 4 Inholland University of Applied Sciences, Amsterdam, the Netherlands; 5 Parnassia Psychiatric Institute, The Hague, the Netherlands; 6 GGZ-VS, Academy for Masters in Advanced Nursing Practice, Utrecht, The Netherlands; 7 Amsterdam Public Health Research Institute (APH), VU University Medical Center, Department of Public and Occupational Health, Amsterdam, the Netherlands; 8 Expertise Center for Palliative Care Amsterdam, VU University Medical Center, Amsterdam, the Netherlands

**Keywords:** Palliative care, Mental healthcare, Severe mental illness, Review

## Abstract

**Background:**

Increasing attention to palliative care for the general population has led to the development of various evidence-based or consensus-based tools and interventions. However, specific tools and interventions are needed for people with severe mental illness (SMI) who have a life-threatening illness. The aim of this systematic review is to summarize the scientific evidence on tools and interventions in palliative care for this group.

**Methods:**

Systematic searches were done in the PubMed, Cochrane Library, CINAHL, PsycINFO and Embase databases, supplemented by reference tracking, searches on the internet with free text terms, and consultations with experts to identify relevant literature. Empirical studies with qualitative, quantitative or mixed-methods designs concerning tools and interventions for use in palliative care for people with SMI were included. Methodological quality was assessed using a critical appraisal instrument for heterogeneous study designs. Stepwise study selection and the assessment of methodological quality were done independently by two review authors.

**Results:**

Four studies were included, reporting on a total of two tools and one multi-component intervention. One study concerned a tool to identify the palliative phase in patients with SMI. This tool appeared to be usable only in people with SMI with a cancer diagnosis. Furthermore, two related studies focused on a tool to involve people with SMI in discussions about medical decisions at the end of life. This tool was assessed as feasible and usable in the target group. One other study concerned the Dutch national Care Standard for palliative care, including a multi-component intervention. The Palliative Care Standard also appeared to be feasible and usable in a mental healthcare setting, but required further tailoring to suit this specific setting. None of the included studies investigated the effects of the tools and interventions on quality of life or quality of care.

**Conclusions:**

Studies of palliative care tools and interventions for people with SMI are scarce. The existent tools and intervention need further development and should be tailored to the care needs and settings of these people. Further research is needed on the feasibility, usability and effects of tools and interventions for palliative care for people with SMI.

**Electronic supplementary material:**

The online version of this article (10.1186/s12888-019-2078-7) contains supplementary material, which is available to authorized users.

## Background

High-quality palliative care is an international human right [1–3]. Every human being deserves access to high-quality palliative care without unnecessary delay. The World Health Organization (WHO) defines palliative care as “an approach that improves the quality of life of patients and their families facing the problem associated with life-threatening illness, through the prevention and relief of suffering by means of early identification and impeccable assessment and treatment of pain and other problems, physical, psychosocial and spiritual” [4].

In the past few decades, palliative care has received increasing attention in policy, practice and research [2, 3]. Yet most attention is given to palliative care for the general population, and less attention has been paid so far to specific groups, such as people with severe mental illness (SMI) [5]. However, it is precisely this group where good and timely palliative care deserves specific attention, because it can be perceived as a particularly vulnerable group. People with SMI are characterized by the presence of persistent psychopathology (> 2 years) and long-term impairments in functioning in multiple areas of life, such as social functioning, education and employment [6–8]. Common diagnoses among this population include psychotic disorders, bipolar disorder, mood/anxiety disorders and severe personality disorders, often accompanied by comorbid addiction disorders [7–10]. Comorbidity of multiple psychiatric and somatic disorders occurs frequently [5, 11–14]. People with SMI have an increased risk of severe co-occurring health problems, such as cardiovascular diseases, neoplasms, respiratory diseases, hepatitis, liver cirrhosis, metabolic syndrome and AIDS [5, 11, 14]. The causes are diverse and often concern a combination of genetic vulnerability, unhealthy lifestyle and the side effects of psychotropic medication.

Under-detection and under-treatment of somatic diseases and symptoms [14], and late access to palliative care is common in people with SMI. This is related to the tendency of people with SMI to avoid professional healthcare [15, 16], and to underreport somatic symptoms, because people with SMI sometimes do not recognize or experience symptoms to the full extent [17]. For example, people with SMI, in particular people with schizophrenia, often perceive less intense pain when compared with the general population [18]. Furthermore, many people with SMI have only a small social network and impaired communication skills, leading to sub-optimal communication with healthcare professionals [15, 17]. Also, psychotic and negative symptoms (e.g. avolition or apathy) may hinder communication about symptoms, problems and palliative care needs. Related to that, people with SMI are less likely to have written advance healthcare directives [19] and are often not involved in decision making about their end-of-life care [20, 21]. All these factors influence the access to and provision of palliative care for people with SMI, and tools and interventions developed for the general palliative care population might therefore not necessarily be suitable for people with SMI.

In addition to these patient-related characteristics, organizational factors in mental healthcare might form a barrier for timely and good palliative care for people with SMI. In the Netherlands and in many other western countries, there is a rather sharp segregation between mental healthcare and somatic healthcare, often leading to poor collaboration between professionals from mental healthcare, general healthcare and palliative care facilities [9, 15, 22–24]. Related to that, mental healthcare organizations often lack expertise in palliative care and the right tools and facilities for such care, particularly where somatic needs are concerned [22].

Several case reports and experts in the mental healthcare field have therefore pointed to the need for tools and interventions that fit with the specific characteristics and settings of people with SMI [25, 26]. There have already been some scoping reviews that show gaps in palliative care for people with SMI [9, 15, 22, 27]. The review presented in this paper now adds to these studies by giving a systematic overview of the available evidence-based tools and interventions for the target group. This paper summarizes the content of evidence-based tools and interventions; it also reports on their usability and feasibility, and whether there is evidence of effects on the quality of life and quality of care.

The following review questions are answered:

1. What tools or interventions are described in empirical research regarding:
  - Identifying and/or discussing the approaching end of life and related palliative care needs;
  - Physical, psychological, social or spiritual care for people with SMI and their relatives?
2. Is there evidence that these tools or interventions are usable and feasible in the palliative care for the target group?
3. Is there evidence that these tools or interventions lead to:
  - A higher quality of life
  - A higher quality of care

for people with SMI and/or their relatives?

With regard to the terms ‘tools’ and ‘interventions’ (see review questions 1, 2 and 3), interventions are regarded in this paper as acts performed by professionals to identify and/or discuss the approaching end of life and related palliative care needs, or to provide palliative care. The execution of an intervention can be supported by a tool (e.g. an instrument, checklist or manual).

With regard to the terms ‘usable’ and ‘feasible’ (see review question 2), we consider a tool or intervention usable when it is perceived as easy or pleasant to work with by professionals and/or patients, and can be applied in palliative care for people with SMI. ‘Feasible’ refers to the extent to which the tool or intervention easily fits into daily routines.

## Methods

### Design

A systematic review of the research literature was carried out to identify relevant studies addressing the topics in our review questions. A review protocol was developed and followed, based on the Preferred Reporting Items for Systematic Reviews and Meta-Analysis (PRISMA) statement [28, 29].

### Inclusion and exclusion criteria

The literature had to meet the following inclusion criteria:

1. Concerns people with SMI and/or their relatives and/or their professional caregivers.
2. Describes the use of tools or interventions for:
  - Identification and/or discussion of the approaching end of life and/or related palliative care needs, and/or
  - Management of physical, psychological, social or spiritual palliative care needs and symptoms.
3. Concerns empirical qualitative, quantitative or mixed-methods research on the effects of the tools and interventions with respect to:
  - Usability and/or feasibility in mental healthcare settings (for inpatient and outpatient care) and/or
  - The quality of care provided to people with SMI in the palliative phase and/or
  - The quality of life during the palliative phase.

Exclusion criteria were:

1. Existent reviews of the literature (although the review reference lists were studied to identify relevant references);
2. Letters to the editors, editorials, congress or conference abstracts, interviews, newspaper articles and comments;
3. Case reports that had not been systematically analysed;
4. Publications focusing on palliative care only in relation to the mental illness.

No period or language restrictions were applied.

### Search strategies and information sources

A comprehensive search was performed in the bibliographic databases PubMed, the Cochrane Library (via Wiley), CINAHL (via Ebsco), PsychINFO (via Ebsco) and Embase.com, from their inception up to 1 June 2017, in collaboration with a medical librarian (LS). Search terms included controlled terms (MesH in PubMed, Emtree in Embase.com etc.) as well as free text terms. We used free text terms only in the Cochrane Library. Search terms expressing ‘palliative care’ were used in combination (AND) with search terms expressing ‘severe mental illness’.

Additionally, we performed reference tracking of the references in the included publications and in some previous literature studies [9, 15, 22, 24, 27]. Furthermore, we searched for additional literature and grey literature by using Google, Google Scholar, OpenGrey, PiCarta and CareSearch with free text terms (e.g. ‘palliative care’, ‘severe mental illness’, ‘terminal’, ‘psychiatry’, ‘research’). Finally, we consulted Dutch experts with expertise regarding palliative care for people with SMI. A full overview of the search strategies used is presented in Additional file 1. All references were entered into EndNote [30], after which duplications in the publications were removed.

### Selection process

Subsequently, all references were independently screened step by step by two authors (KB and AF), with the aid of the software tool Covidence (https://www.covidence.org/). In the first step, the authors (KB and AF) independently screened titles and abstracts to identify potentially relevant publications. They subsequently compared and discussed their selections. In the following step, the full texts of the remaining publications were assessed by both authors (KB and AF). Then disagreements were resolved by discussion between the two authors (KB and AF).

### Data extraction

The data on the included studies were extracted using a pre-structured form (see Additional file 2), which was developed by the authors (KB and AF). Two authors (KB and BM) extracted the data independently and compared their ratings. In the event of any disagreements, a third researcher could be consulted. Consultation was not needed since there was full agreement between the two authors at this stage.

The extracted data included characteristics of the study design, data collection, characteristics of the participants, results (ordered on the basis of the research questions), and the outcomes of the methodological appraisal.

### Critical appraisal of the methodological quality

Because of the heterogeneous research methods applied in the studies (quantitative, qualitative or mixed methods), we used the critical appraisal instrument of Hawker et al. [31] to assess methodological quality. This instrument is appropriate for appraising studies with different research methods. It has proven to be useful in various other systematic reviews for assessing the quality of studies with heterogeneous designs [32–35]. The instrument appraises the following nine elements: abstract, background, methodology, sampling, data analysis, ethics, results, transferability and implications. Each element is scored on a four-point scale, ranging from very poor to good. The total score can range from 9 to 36; scores less than or equal to 18 are rated as ‘poor quality’, scores from 19 to 27 as ‘fair quality’ and above 27 as ‘good quality’. Two reviewers (KB and BM) conducted the methodological assessments independently and agreed to use a threshold of up to five points. If the methodological quality scores differed by more than five points, the average of the scores was calculated. In practice, this did not occur.

## Results

### Process for selecting the studies

The various search strategies (see above) resulted in a total of 2359 unique references.

Twenty-seven publications remained after the first selection step (based on reading titles and abstracts). The full texts were then screened, after which 23 publications were excluded [36–55]. The reasons for exclusion are presented in Additional file 3.

The two-step selection process ultimately resulted in four included studies. The results of the selection process are presented in the PRISMA Flow Chart in Additional file 4.

### Characteristics of the included studies

Table 1 shows the aims and methodological characteristics (study design, population and data collection) of the studies.

**Table 1:** Aims and methodological characteristics of the included studies

Authors Date Country	Aim	Study design/ population	Data collection
Burton et al. 2016 USA [56]	Examine the validity of the CARING criteria for adults admitted to an inpatient psychiatric unit.	Quantitative retrospective design / Inpatients at an acute psychiatric unit (*n* = 276).	1) 276 medical records 2) Data included: Demographic information; CARING criteria including National Hospice and Palliative Care Organization (NHPCO) non-cancer hospice guidelines; ICU admission with multi-organ failure, and mortality within 1 year of index hospitalization; date of death.
Foti et al. 2005 USA [57]	Examine preferences regarding advance healthcare planning among persons with SMI, specifically, experience, beliefs, values and concerns about healthcare proxies and end-of-life issues	Mixed-methods design / 150 patients with SMI who received mental health services. They had at least one medical diagnosis.	1) Data form: sociodemographic characteristics, psychiatric diagnoses (DSM-IV), medication, medical conditions, frequency of medical specialty visits or hospitalizations, and Current Evaluation of Risk and Functioning-Revised (CERF-R) scores. 2) Structured interview with the Health Care Preferences Questionnaire (HCPQ). 3) Interview feedback + follow-up
Foti et al. 2005 USA [58]	Ascertain preferences for end-of-life care in relation to various hypothetical medical health state scenarios among persons with SMI.	Mixed-methods design / 150 patients with SMI who received mental health services. They had at least one medical diagnosis.	1) Structured interview with the Health Care Preferences Questionnaire (HCPQ) supplemented with two hypothetical health state scenarios, and questions derived from the Quest to Die With Dignity. 2) Interview feedback + follow-up
Smits et al. 2015 The Netherlands [59]	Examine the following questions: 1) Is the pilot implementation of the Palliative Care Standard perceived as useful and usable by the participants? 2) Is the Palliative Care Standard usable for contracting policies by the health insurance companies? 3) Are the recommendations in the Care Standard feasible in clinical practice? 4) Is the Palliative Care Standard usable for specific target groups (e.g. people with a psychiatric illness)?	Mixed-methods design / 105 participants, including project leaders of the pilot implementation, care professionals, patient representatives, informal caregivers and managers from seven different healthcare settings (e.g. mental healthcare, general hospital care, hospice care, general practices)	• Questionnaires • Interviews • Several focus group discussions in which findings and opinions about the pilot implementation were examined

One of the four studies, namely that of Burton et al. [56], focused on the validity of an identification tool for identifying that death is approaching in people who are admitted to an inpatient psychiatric unit. Two related studies, namely those by Foti et al. [57, 58], concerned one and the same tool, namely a tool for discussing end-of-life treatment preferences and medical decisions for people with SMI.

The fourth study, performed by Smits et al. [59], examined the applicability of the Dutch national Palliative Care Standard (in Dutch: *zorgmodule palliatieve zorg*) within various healthcare settings, including a mental healthcare organization.

In the study by Burton et al. [56] the medical records were analysed, while in the two studies by Foti et al. [57, 58] structured interviews were performed with patients with SMI. In addition, Foti et al. used the Current Evaluation of Risk and Functioning-Revised (CERF-R) tool to collect data about risk, functioning and the level of care needs directly related to the psychiatric diagnosis.

In the study by Smits et al. [59], data were collected via questionnaires, interviews and focus-group discussions with project managers, healthcare professionals and patient representatives. The methodological quality of the studies (Table 2) varied from poor [59] and fair [56] to good [57, 58].

**Table 2:** Outcomes of the methodological appraisal

	Burton, 2016 [56]	Foti, 2005 [57]	Foti, 2005 [58]	Smits, 2015 [59]
Abstract & title	Poor 2	Fair 3	Fair 3	Poor 2
Introduction & aims	Fair 3	Good 4	Good 4	Poor 2
Method & data	Fair 3	Fair 3	Fair 3	Poor 2
Sampling	Poor 2	Good 4	Fair 3	Poor 2
Data analysis	Poor 2	Fair 3	Good 4	Very poor 1
Ethics & bias	Very poor 1	Very poor 1	Very poor 1	Very poor 1
Results & findings	Fair 3	Fair 3	Fair 3	Poor 2
Transferability & generalizability	Poor 2	Good 4	Good 4	Fair 3
Implications	Poor 2	Fair 3	Fair 3	Fair 3
Total score/ quality	20 ‘Fair’	28 ‘Good’	28 ‘Good’	18 ‘Poor’

### Description of the tools and interventions for palliative care

The first research question addressed the available empirically studied tools and interventions that can be used in palliative care for people with SMI and their relatives. The four studies described a total of two tools and one multi-component intervention for palliative care (Table 3). These concerned:

1. A tool to identify patients who might need palliative care [56]
2. A tool to talk about medical decisions that must be made with respect to the care needs and preferences of the patient and their relatives [57, 58]
3. A national Palliative Care Standard describing a multi-component intervention for providing good quality palliative care [59].

**Table 3:** Characteristics of the tools and intervention

Study reference	
Burton, 2016 [56]	The CARING criteria: a set of prognostic criteria to identify persons near the end of life upon hospital admission. It has five Indicators:
	• Cancer as the primary diagnosis,
	• Admissions: twice or more in the past year for a chronic illness,
	• Residence in a nursing home,
	• Intensive Care Unit (ICU) admission with multi-organ failure,
	• Non-cancer Hospice Guidelines
	The CARING criteria must be applied to patients who are hospitalized on the first day after admission.
Foti, 2005 [57]	Current Evaluation of Risk and Functioning-Revised (CERF-R): An 18-item scale assessing client functioning and risk (functional disability), Short Form-12 (SF-12): assesses Health care status. It contains 12 questions concerning:
	• Physical functioning,
	• Role limitations because of physical health problems
	• Bodily pain
	• General health perception
	• Vitality (energy/ fatigue)
	• Social functioning
	• Role limitations because of emotional problems
	• General mental health (psychological distress and psychological well-being)
	Health Care Preferences Questionnaire (HCPQ). This questionnaire documents attitudes and preferences for scenario-based choices and was used for advance healthcare planning. HCPQ components include:
	• Health status, assessed with the SF-12
	• Advance care planning
	• Scenario-based treatment preferences
	• Beliefs, values and concerns about the end of life. Interview feedback and follow-up
	• Interviewer’s addendum.
	Designation of a healthcare proxy (a relative or professional) to make healthcare decisions for a person who is not able to do so.
Foti, 2005 [58]	Health Care Preferences Questionnaire (HCPQ): see full description above. The HCPQ also contains a psychiatric health state scenario. Two hypothetical health state scenarios supplemented with questions derived from the Quest to Die with Dignity instrument
Smits, 2015 [59]	The Dutch Palliative Care Standard describes six building blocks, namely:
	• Vision and policy
	• ‘Surprise Question’ to identify approaching death
	• Use of an ‘Individual Care Plan’ and ‘Shared Decision Making’ within the framework of ‘Advance Care Planning’
	• Expertise for delivering high-quality palliative care
	• The organization of palliative care
	• Quality indicators

The two tools and the Palliative Care Standard are described below and the characteristics are shown in Table 3.

The first tool, described in the study by Burton et al. [56], is intended for the identification of patients who might need palliative care. The tool is based on the CARING criteria, which are applied to people admitted to a psychiatric unit [56]. The CARING criteria are a set of five prognostic criteria that have been proposed for the identification of persons who are near the end of life upon hospital admission. The five indicators are **C**ancer as the primary diagnosis, **A**dmitted at least two times in the past year for a chronic illness, **R**esident in a nursing home and **I**ntensive Care Unit admission due to multi-organ failure. A **N**on-cancer hospice **G**uideline is used for the fifth criterion, which is scored depending on the nature of the main problem (e.g. renal, cardiac or liver). The objective of the study by Burton et al. was to examine the validity of these criteria when applied to patients admitted to an acute inpatient psychiatric unit after being referred from an academic tertiary care centre.

The second tool, described in the two studies by Foti et al. [57, 58], is the Health Care Preferences Questionnaire (HCPQ). The HCPQ provides information for advance care planning and end-of-life preferences of people with SMI [57, 58]. Two hypothetical scenarios are used to assess a person’s advance care planning preferences [58]. These scenarios present patients who have conditions that prevent them from expressing a choice between different types of palliative care, aggressive treatments for their somatic condition and life support. The two scenarios concern a patient with terminal metastatic cancer who is in terrible pain and a patient with total paralysis and irreparable brain damage. Other examples of topics in the HCPQ are previous experiences with advance care planning and who should be designated as a proxy to make healthcare decisions for a person who is too sick to do. In this study, the HCPQ was administered by trained interviewers.

Thirdly, the Dutch national Palliative Care Standard described in the study by Smits et al. [59] defines high-quality palliative care and can be used by professionals and organizations providing palliative care in various healthcare settings. The Palliative Care Standard contains six ‘building blocks’:

1. Vision and policy on palliative care. This section is about the need to have a vision that enjoys broad support and which should be translated into specific policies and agreements to provide high-quality palliative care.
2. Identification of the palliative phase. In the Palliative Care Standard, the ‘Surprise Question’ is recommended to identify the palliative phase. The Surprise Question is a question designed to determine a possible approaching death: “Would I be surprised if this patient died in the next twelve months?” If the answer is “no”, palliative care may be needed.
3. The Individual Care Plan. This plan organizes and supports the care process, and covers physical, psychological, social and spiritual needs. Collaborative advance care planning with the patient and relatives is a key element. The Individual Care Plan should support an integrative and multidisciplinary care approach.
4. Expertise for delivering high-quality palliative care during every stage at the end of life. Providing high-quality palliative care requires specific attitudes, knowledge and skills.
5. The organization of palliative care. This building block describes a framework for organizing flexible high-quality palliative.
6. Quality indicators. In this building block, measurable quality indicators are presented for several areas (e.g. the presence of a care plan or care for bereaved relatives).

### Usability and feasibility of the tools and interventions

The second research question concerns the empirical evidence on the usability and feasibility of the tools and intervention in palliative care for people with SMI. Table 4 summarizes the results regarding usability and feasibility.

**Table 4:** Usability and feasibility of the tools and intervention

Study reference	Tool or intervention	Usability or feasibility
Burton, 2016 [56]	• CARING criteria are used as a set of prognostic criteria that have been proposed for identification of persons near the end of life upon hospital admission.	Applying the CARING criteria was problematic in a patient population with psychiatric disorders where cancer will almost never be the primary diagnosis.
Foti, 2005 [57]	• Advance healthcare planning through a structured interview using the Health Care Preferences Questionnaire (HCPQ). • Healthcare proxy designation	1) Advance healthcare planning with semi-structured interviews such as the HCPQ suggests a standardized approach to advance healthcare planning for people with SMI is feasible and acceptable. 2) Healthcare proxy designation and end-of-life care concerns can be ascertained through a semi-structured interview conducted by mental health providers without adverse effects. The HCPQ is usable within the target group.
Foti, 2005 [58]	Advance care planning through the HCPQ (see Foti above). Supplemented with two hypothetical health state scenarios, derived from the Quest to Die With Dignity.	1) Mental health consumers were able to engage in advance healthcare planning through hypothetical health state preference scenarios. Obtaining healthcare preferences by using hypothetical scenarios is feasible. 2) The HCPQ with hypothetical scenarios was usable for people with SMI, although some questions in the interview distressed participants.
Smits, 2015 [59]	The Palliative Care Standard consists of six building blocks. The Palliative Care Standard covers identification of the palliative phase using the Surprise Question, Advance Care Planning, Individual Care Plan, Shared Decision Making and Quality Indicators for palliative care.	The Palliative Care Standard was usable and feasible, including in a setting for people with a psychiatric disorder. However, recommendations need to be tailored to better suit the specific target groups.

The study by Burton et al. [56] concludes that the CARING criteria are only partly usable when it comes to people admitted to a psychiatric hospital. Burton et al. conclude that appropriate criteria for identifying people who are near the end of life are: two or more psychiatric admissions in the previous year, advanced age and residence in a nursing home before admission. In addition, the authors suggest taking premorbid dementia into account [56].

The two studies by Foti et al. [57, 58] conclude that people with SMI are able to respond to difficult end-of-life questions and that the HCPQ is a usable tool for talking with them about advance care planning at the end of life. The average duration of the interview guided by the HCPQ was about 45 min, which was not considered problematic. Nearly all (96%) of the participants with SMI completed the interview; a small majority (58%) were comfortable with the interview and most participants (77%) were willing to participate in a follow-up interview [57]. Based on these results, Foti et al. [58] concluded that the HCPQ seemed to be usable and feasible for this patient group.

The study by Smits et al. [59] showed that the Dutch Palliative Care Standard could be used for different target groups, including people with a chronic psychiatric disorder. The mental healthcare professionals were initially sceptical when starting to work with this Care Standard, but eventually they found it usable. Nevertheless, Smits et al. concluded that the general recommendations in the standard need to be tailored to better suit the characteristics of the specific target group. The authors also recommend paying extra attention to situations where people cannot properly express their needs and wishes, and are less able to participate in shared decision making. However, Smits et al. concluded that the main elements of the Palliative Care Standard, such as the Surprise Question, are usable and feasible in palliative care for people with SMI.

### Effects of the tools and interventions on quality of life or quality of care

None of the studies included in this review investigated the effect of the relevant tool or intervention on quality of life or quality of care (i.e. research question 3).

## Discussion

This systematic review focused on empirically based tools and interventions that can be used in palliative care for people with SMI. The first research question addressed the kinds of tools and interventions used for palliative care. Only four relevant empirical studies were found. They covered two tools and one multi-component intervention: a tool for the early identification of palliative care needs [56], a tool to discuss medical decisions and end-of-life care planning with the patient and relatives [57, 58] and a multi-component Care Standard describing building blocks for high-quality palliative care [59]. The paucity of interventions and the scarcity of empirical studies addressing palliative care for people with SMI were also mentioned in earlier studies [9, 22, 24].

The feasibility and usability (research question 2) were discussed in all four studies, although this was often not the primarily focus of the study. The tool for discussing medical decisions [57, 58] and the Care Standard [59] were found to be feasible and usable. The tool for identifying palliative care needs [56] appeared not to be usable (and thus also not feasible) in palliative care for patients with SMI, and major adaptions were suggested by the researchers.

None of the included studies examined the effects that the use of the tools or interventions had on the quality of life of patients or on the quality of care. This fits with the conclusions in systematic reviews of palliative care tools or interventions for other specific target groups, such as people with intellectual disabilities or homeless people [32].

Despite the very limited evidence base, we can conclude that at least some tools and interventions for palliative care in people with SMI exist. The tool described by Burton et al. [56] addresses criteria for the timely identification of approaching death in patients with SMI. Use of this tool might make professionals more alert to the possibility that a person is in the palliative phase [56]. However, the tool addresses a very specific group (persons with SMI and cancer), and some of the criteria that are part of this tool would probably have to be adapted for use in other palliative-care patients with SMI.

The second tool, investigated by Foti et al. [57, 58], aims to involve patients with SMI in advance care planning, which is found to be feasible. Discussing medical decisions with people with SMI did not result in severe distress [58]. This was also found in a review [60] of mental health advance treatment directives for people with SMI who might become unable to adequately express their mental health treatment preferences in the future due to acute symptoms of their psychiatric disorder. However, this review found little data indicating that mental health advance treatment directives lead to more favourable healthcare outcomes.

The third intervention, described by Smits et al. [59], concerns a multi-component intervention, the Palliative Care Standard. This Care Standard is found to be useful for formulating an intervention programme to improve palliative care for people with SMI, although adjustments and tailoring to suit the specific characteristics of the patient population and the mental healthcare setting were found to be necessary [59]. Previous publications primarily focused on whether current palliative care is already tailored to the needs of people with SMI [9, 15, 22, 24].

### Strengths and limitations

A strength of this systematic review is the focus on empirically based information about the availability, usability, feasibility and effectiveness of tools and interventions within palliative care for people with SMI. To the best of our knowledge, this is the first review to have investigated this issue. The results can be considered as an important step towards more empirically based, effective palliative care for people with SMI. A limitation is that only a few empirical studies were found, dealing with diverse tools and interventions, which reduces the generalizability of the results.

### Implications for practice and further research

Based on this systematic review, we recommend the following tools and interventions for palliative care for people with SMI in mental health practice. Firstly, palliative care is complex and not usually part of daily mental healthcare. The multi-component Care Standard can be of great added value as it provides well-defined recommendations for policy and practice to improve palliative care.

Secondly, timely identification of the palliative phase is needed so that medical needs and preferences can be discussed in good time and high-quality palliative care provided. The Surprise Question is a tool for identifying the palliative phase. It could be adopted in a regular annual or half-yearly evaluation of the treatment plan as a minimum, or in addition to the annual somatic screening. In addition to the Surprise Question, the CARING criteria can be used to help identify the palliative phase.

Thirdly, we recommend a proactive palliative approach through timely discussion of end-of-life care preferences and medical decisions. Hypothetical scenarios could be helpful in examining these preferences and arriving at medical care decisions among people with SMI. Timely discussion and accurate documentation of medical healthcare decisions and preferences can lead to better access to palliative services and a higher quality of life in the palliative phase.

Fourthly, mental healthcare professionals often see people with SMI as too vulnerable or incompetent to discuss their approaching death and end-of-life decisions. However, people with SMI often do have the capacity to discuss their medical preferences and appear to be interested in doing so. Shared decision making perhaps implies a need for better communication skills among healthcare professionals [19, 24, 58].

In addition, it is important that providers of mental healthcare and providers of palliative care engage in a partnership. This will enable knowledge and experiences to be exchanged (e.g. cross-training) and collaborative care to be delivered [15, 24]. Based on the results of this systematic review and in particular the knowledge gaps we found, the following questions for future research should be prioritized:

1. What tools and interventions for high-quality palliative care for patients with SMI can be developed, building further on the few available tools and interventions or elements thereof?
2. What barriers and facilitators can be identified in the implementation of these tools and interventions at the various interacting levels of the patient, relatives, professionals and organizations? And how can these barriers and facilitators be addressed?
3. How effective are these tools and interventions with respect to the quality of care and quality of life of patients from this target group?

## Conclusions

Two empirically based tools and one multi-component intervention are available for improving palliative care for this group. However, these tools and this intervention need further development. They should be better tailored to the specific care needs of people with SMI, and it should be made easier for care professionals to apply them in mental healthcare settings.

## Additional files

Additional file 1: Search strategies. Full overview of the search strategies. (DOCX 21 kb)

Additional file 2: Data extraction form. Presents the pre-structured form, which is used for data extraction of the included studies. (DOCX 17 kb)

Additional file 3: Reasons for exclusion full text screening. Overview of the reasons for exclusion of the full text screening step. (DOCX 32 kb)

Additional file 4: PRISMA flow chart. Full overview of selection process in PRISMA flow chart. (DOCX 121 kb)

## Abbreviations

AF: Anneke Francke
AV: Anke de Veer
BM: Berno van Meijel
CERF-R: Current Evaluation of Risk and Functioning-Revised
HCPQ: Health Care Preferences Questionnaire
ICU: Intensive Care Unit
KB: Karin den Boer
KV: Kim Verhaegh
LS: Linda Schoonmade
PRISMA: Preferred Reporting Items for Systematic Reviews and Meta-Analysis
SF-12: Short Form-12
SMI: Severe mental illness
