# Supplementary material for: A systematic review of palliative care tools and interventions for people with severe mental illness
Source: BMC Psychiatry. 2019 Apr 3;19:106. doi: 10.1186/s12888-019-2078-7 (PMC6446277; doi:10.1186/s12888-019-2078-7)
Supplement: Supplementary file 1 — Search strategies. Full overview of the search strategies. (DOCX 21 kb) [file 12888_2019_2078_MOESM1_ESM.docx]

**Additional File 1** **Search strategies**

## Pubmed history, 1 June 2017

| Search | Query | Results |
| --- | --- | --- |
| #4 | #3 NOT ("Addresses"[Publication Type] or "Biography"[Publication Type] or "Comment"[Publication Type] or "Directory"[Publication Type] or "Editorial"[Publication Type] or "Festschrift"[Publication Type] or "Interview"[Publication Type] or "Lectures"[Publication Type] or "Legal Cases"[Publication Type] or "Legislation"[Publication Type] or "Letter"[Publication Type] or "News"[Publication Type] or "Newspaper Article"[Publication Type] or "Patient Education Handout"[Publication Type] or "Popular Works"[Publication Type] or "Congresses"[Publication Type] or "Consensus Development Conference"[Publication Type] or "Consensus Development Conference, Nih"[Publication Type]) | 913 |
| #3 | #1 AND #2 | 970 |
| #2 | "Bipolar Disorder"[Mesh] OR "Affective Disorders, Psychotic"[Mesh] OR "Depressive Disorder, Major"[Mesh] OR "Paranoid Disorders"[Mesh] OR "Psychotic Disorders"[Mesh] OR "Schizophrenia"[Mesh] OR "Schizophrenia Spectrum and Other Psychotic Disorders"[Mesh] OR "Mentally Ill Persons"[Mesh] OR bipolar disorder*[tiab] OR manic-depress*[tiab] OR mania*[tiab] OR psychotic affective disorder*[tiab] OR affective psychos*[tiab] OR psychotic mood disorder*[tiab] OR psychotic depress*[tiab] OR schizoaffective disorder*[tiab] OR paranoid disorder*[tiab] OR psychotic disorder*[tiab] OR schizophreni*[tiab] OR severe psychiatric ill*[tiab] OR serious psychiatric ill*[tiab] OR chronic psychiatric ill*[tiab] OR persistent psychiatric ill*[tiab] OR severe mental ill*[tiab] OR serious mental ill*[tiab] OR chronic mental ill*[tiab] OR persistent mental ill*[tiab] OR serious mentally ill*[tiab] OR severe mentally ill*[tiab] OR chronic mentally ill*[tiab] OR persistent mentally ill*[tiab] OR severe psychiatric disorder*[tiab] OR serious psychiatric disorder*[tiab] OR chronic psychiatric disorder*[tiab] OR persistent psychiatric disorder*[tiab] OR severe mental disorder*[tiab] OR serious mental disorder*[tiab] OR persistent mental disorder*[tiab] OR chronic mental disorder*[tiab] | 228556 |
| #1 | "Palliative Care"[Mesh] OR "Hospice Care"[Mesh] OR "Terminal Care"[Mesh] OR "Terminally Ill"[Mesh] OR palliati*[tiab] OR terminal[tiab] OR terminally ill*[tiab] OR end stage disease*[tiab] OR end-of-life*[tiab] OR hospice*[tiab] | 485636 |

## Embase history, 1 June 2017

| Search | Query | Results |
| --- | --- | --- |
| #4 | #3 NOT ('conference abstract'/it OR 'conference review'/it OR 'editorial'/it OR 'erratum'/it OR 'letter'/it OR 'note'/it) | 1077 |
| #3 | #1 AND #2 | 1439 |
| #2 | 'bipolar disorder'/exp OR 'affective psychosis'/exp OR 'major depression'/exp OR 'paranoid psychosis'/exp OR 'endogenous psychosis'/exp OR 'manic psychosis'/exp OR 'schizophrenia'/exp OR 'mental patient'/exp OR 'bipolar disorder*':ab,ti OR 'manic-depress*':ab,ti OR mania*:ab,ti OR (psychotic NEAR/3 disorder*):ab,ti OR 'affective psychos*':ab,ti OR 'psychotic depress*':ab,ti OR (schizoaffective NEAR/3 disorder*):ab,ti OR (paranoid NEAR/3 disorder*):ab,ti OR schizophren*:ab,ti OR 'severe psychiatric ill*':ab,ti OR 'serious psychiatric ill*':ab,ti OR 'chronic psychiatric ill*':ab,ti OR 'persistent psychiatric ill*':ab,ti OR 'severe mental* ill*':ab,ti OR 'serious mental* ill*':ab,ti OR 'chronic mental* ill*':ab,ti OR 'persistent mental* ill*':ab,ti OR 'severe psychiatric disorder*':ab,ti OR 'serious psychiatric disorder*':ab,ti OR 'chronic psychiatric disorder*':ab,ti OR 'persistent psychiatric disorder*':ab,ti OR 'severe mental* disorder*':ab,ti OR 'serious mental disorder*':ab,ti OR 'persistent mental disorder*':ab,ti OR 'chronic mental disorder*':ab,ti | 307946 |
| #1 | 'terminal care'/de OR 'hospice care'/exp OR 'palliative nursing'/exp OR 'terminally ill patient'/exp OR palliati*:ab,ti OR terminal:ab,ti OR 'terminally ill*':ab,ti OR 'end stage disease*':ab,ti OR 'end of life':ab,ti OR hospice*:ab,ti | 530270 |

## Cinahl (Ebsco) history, 1 June 2017

| Search | Query | Results |
| --- | --- | --- |
| #3 | #1 AND #2 | 152 |
| #2 | MH ("Psychotic Disorders" OR "Affective Disorders, Psychotic" OR "Bipolar Disorder" OR "Paranoid Disorders" OR "Schizoaffective Disorder" OR "Schizophrenia" OR "Psychiatric Patients" OR "Mental Disorders, Chronic") OR TI (“bipolar disorder*” OR “manic-depress*” OR mania* OR psychotic N3 disorder* OR “affective psychos*” OR “psychotic depress*” OR schizoaffective N3 disorder* OR paranoid N3 disorder* OR schizophren* OR “severe psychiatric ill*” OR “serious psychiatric ill*” OR “chronic psychiatric ill*” OR “persistent psychiatric ill*” OR “severe mental* ill*” OR “serious mental* ill*” OR “chronic mental* ill*” OR “persistent mental* ill*” OR “severe psychiatric disorder*” OR “serious psychiatric disorder*” OR “chronic psychiatric disorder*” OR “persistent psychiatric disorder*” OR “severe mental* disorder*” OR “serious mental disorder*” OR “persistent mental disorder*” OR “chronic mental disorder*”) OR AB (“bipolar disorder*” OR “manic-depress*” OR mania* OR psychotic N3 disorder* OR “affective psychos*” OR “psychotic depress*” OR schizoaffective N3 disorder* OR paranoid N3 disorder* OR schizophren* OR “severe psychiatric ill*” OR “serious psychiatric ill*” OR “chronic psychiatric ill*” OR “persistent psychiatric ill*” OR “severe mental* ill*” OR “serious mental* ill*” OR “chronic mental* ill*” OR “persistent mental* ill*” OR “severe psychiatric disorder*” OR “serious psychiatric disorder*” OR “chronic psychiatric disorder*” OR “persistent psychiatric disorder*” OR “severe mental* disorder*” OR “serious mental disorder*” OR “persistent mental disorder*” OR “chronic mental disorder*”) | 32,166 |
| #1 | MH ("Terminal Care" OR "Palliative Care" OR "Hospice Care" OR "Terminally Ill Patients" OR "Hospice Patients") OR TI (palliati* OR terminal OR “terminally ill*” OR “end stage disease*” OR “end of life” OR hospice*) OR AB (palliati* OR terminal OR “terminally ill*” OR “end stage disease*” OR “end of life” OR hospice*) | 56,901 |

## PsycINFO (Ebsco) history, 1 June 2017

| Search | Query | Results |
| --- | --- | --- |
| #4 | S3 NOT (ZZ "abstract collection") or (ZZ "bibliography") or (ZZ "clarification") or (ZZ "column/opinion") or (ZZ "comment/reply") or (ZZ "editorial") or (ZZ "encyclopedia entry") or (ZZ "erratum/correction") or (ZZ "interview") or (ZZ "letter") or (ZZ "obituary") or (ZZ "poetry") or (ZZ "publication information") or (ZZ "review-book") or (ZZ "review-media") or (ZZ "review-software & other") | 328,851 |
| #3 | S1 AND S2 | 1,182 |
| #2 | DE ("Bipolar Disorder" OR "Affective Psychosis" OR "Major Depression" OR "Mania" OR "Schizoaffective Disorder" OR "Chronic Psychosis" OR "Schizophrenia" OR "Paranoia (Psychosis)" OR "Paranoid Schizophrenia" OR "Psychiatric Patients" OR "Chronic Mental Illness") OR TI (“bipolar disorder*” OR “manic-depress*” OR mania* OR (psychotic N3 disorder*) OR “affective psychos*” OR “psychotic depress*” OR (schizoaffective N3 disorder*) OR (paranoid N3 disorder*) OR schizophren* OR “severe psychiatric ill*” OR “serious psychiatric ill*” OR “chronic psychiatric ill*” OR “persistent psychiatric ill*” OR “severe mental* ill*” OR “serious mental* ill*” OR “chronic mental* ill*” OR “persistent mental* ill*” OR “severe psychiatric disorder*” OR “serious psychiatric disorder*” OR “chronic psychiatric disorder*” OR “persistent psychiatric disorder*” OR “severe mental* disorder*” OR “serious mental disorder*” OR “persistent mental disorder*” OR “chronic mental disorder*”) OR AB (“bipolar disorder*” OR “manic-depress*” OR mania* OR (psychotic N3 disorder*) OR “affective psychos*” OR “psychotic depress*” OR (schizoaffective N3 disorder*) OR (paranoid N3 disorder*) OR schizophren* OR “severe psychiatric ill*” OR “serious psychiatric ill*” OR “chronic psychiatric ill*” OR “persistent psychiatric ill*” OR “severe mental* ill*” OR “serious mental* ill*” OR “chronic mental* ill*” OR “persistent mental* ill*” OR “severe psychiatric disorder*” OR “serious psychiatric disorder*” OR “chronic psychiatric disorder*” OR “persistent psychiatric disorder*” OR “severe mental* disorder*” OR “serious mental disorder*” OR “persistent mental disorder*” OR “chronic mental disorder*”) | 276,740 |
| #1 | DE ("Palliative Care" OR "Hospice" OR "Terminally Ill Patients") OR TI (palliati* OR terminal OR “terminally ill*” OR “end stage disease*” OR “end of life” OR hospice*) OR AB (palliati* OR terminal OR “terminally ill*” OR “end stage disease*” OR “end of life” OR hospice*) | 36,748 |

## The Cochrane Library (Wiley) history, 1 June 2017

| Search | Query | Results |
| --- | --- | --- |
| #3 | #1 AND #2 | 43 |
| #2 | "bipolar disorder*" or "manic-depress*" or mania* or (psychotic near/3 disorder*) or "affective psychos*" or "psychotic depress*" or (schizoaffective near/3 disorder*) or (paranoid near/3 disorder*) or schizophren* or "severe psychiatric ill*" or "serious psychiatric ill*" or "chronic psychiatric ill*" or "persistent psychiatric ill*" or "severe mental* ill*" or "serious mental* ill*" or "chronic mental* ill*" or "persistent mental* ill*" or "severe psychiatric disorder*" or "serious psychiatric disorder*" or "chronic psychiatric disorder*" or "persistent psychiatric disorder*" or "severe mental* disorder*" or "serious mental disorder*" or "persistent mental disorder*" or "chronic mental disorder*":ti,ab,kw (Word variations have been searched) | 17480 |
| #1 | palliati* or terminal or "terminally ill*" or "end stage disease*" or "end of life" or hospice*:ti,ab,kw (Word variations have been searched) | 11471 |
