# Supplementary material for: A systematic review of palliative care tools and interventions for people with severe mental illness
Source: BMC Psychiatry. 2019 Apr 3;19:106. doi: 10.1186/s12888-019-2078-7 (PMC6446277; doi:10.1186/s12888-019-2078-7)
Supplement: Supplementary file 2 — Data extraction form. Presents the pre-structured form, which is used for data extraction of the included studies. (DOCX 17 kb) [file 12888_2019_2078_MOESM2_ESM.docx]

**Additional file 2**

**Data extraction form**

1. SMI study:

- Reference: authors/date/title
- Country
- Aim and/or research questions

1. Study design and data collection:

- Quantitative: survey, RCT, CCT, cohort study, cross-sectional study Qualitative: interviews, observations, qualitative document analyses, …….
- Total number of participants (n)
- Sources: mental healthcare professionals (medical doctor, general practitioner, nurse, psychiatrist, psychologist, nurse practitioner, social worker, counsellor, therapist, pastoral counsellor, peer specialist), people with SMI, relatives, client files/registrations, others

1. Background information on people with SMI:

- Age category
- Type of (pre-existing) severe mental illness
- Types of somatic illness
- Setting

1. Results:

- Answers to research questions
- Content of tool or intervention for:
  - identification of /communication about approaching death and/or palliative care needs
  - management of physical, psychological, social or spiritual palliative care needs
  - management of physical, psychological, social or spiritual symptoms
- Effects on
  - (aspects of) quality of life
  - (aspects of) quality of care
  - outcomes regarding usability or feasibility
- Main conclusion of authors

1. Methodological quality
   - Abstract and title
   - Introduction and aims
   - Data analysis
   - Result and findings
   - Transferability or generalizability
   - Implications and usefulness
   - Total score Critical Appraisal Tool
   - Strengths of design
   - Limitations of design
