# Supplementary material for: A systematic review of palliative care tools and interventions for people with severe mental illness
Source: BMC Psychiatry. 2019 Apr 3;19:106. doi: 10.1186/s12888-019-2078-7 (PMC6446277; doi:10.1186/s12888-019-2078-7)
Supplement: Supplementary file 3 — Reasons for exclusion full text screening. Overview of the reasons for exclusion of the full text screening step. (DOCX 32 kb) [file 12888_2019_2078_MOESM3_ESM.docx]

**Additional file 3 Reasons for exclusion full text screening**

| **Publication** | **Excluded with reasons** |
| --- | --- |
| [41] 2009 | Letter/ editorial/ congress abstract/ interview/ newspaper article/ comment |
| [42] 2000 Addington-Hall et al. | Not an empirical qualitative, quantitative, mixed methods research |
| [43] 1996 Akamatsu | Letter/ editorial/ congress abstract/ interview/ newspaper article/ comment |
| [44] 2012 Avery et al. | Not systematically analysed case report |
| [45] 2000 Bakker | Does not concern people with severe mental illness/relatives/ their professional caregivers |
| [46] 2012 Berk et al. | Review |
| [47] Berlim et al.2005 | No method, tool or intervention for identification/discussion/ management of nearing death/ palliative care needs/symptoms |
| [40] Campbell et al. | Review |
| [48] 1999 Candilis et al. | Not an empirical qualitative, quantitative, mixed methods research |
| [21] 2004 Candilis et al. | Not an empirical qualitative, quantitative, mixed methods research |
| [49] 2006 Davie | Full text not found |
| [25] 2015 Ganzini | Not an empirical qualitative, quantitative, mixed methods research |
| [50] 2017 Harman | Not an empirical qualitative, quantitative, mixed methods research |
| [51] 1999 Hilderley et al. | No method, tool or intervention for identification/discussion/ management of nearing death/ palliative care needs/symptoms |
| [52] 1999 Lent et al. | Not an empirical qualitative, quantitative, mixed methods research |
| [53] 2009 Moorey et al. | Does not concern people with severe mental illness/relatives/ their professional caregivers |
| [54] 2011 O’Neill | No method, tool or intervention for identification/discussion/ management of nearing death/ palliative care needs/symptoms |
| [55] 2002 Papageorgio et al. | No method, tool or intervention for identification/discussion/ management of nearing death/ palliative care needs/symptoms |
| [56] 2015 Picot et al. | Not an empirical qualitative, quantitative, mixed methods research |
| [57] 2013 Rhondali et al. | No method, tool or intervention for identification/discussion/ management of nearing death/ palliative care needs/symptoms |
| [58] 1993 Stecker | No method, tool or intervention for identification/discussion/ management of nearing death/ palliative care needs/symptoms |
| [59] 2005 Tate et al. | Not an empirical qualitative, quantitative, mixed methods research |
| [60] 2014 Terpstra et al. | Not an empirical qualitative, quantitative, mixed methods research |
