# Supplementary material for: A systematic review of palliative care tools and interventions for people with severe mental illness
Source: BMC Psychiatry. 2019 Apr 3;19:106. doi: 10.1186/s12888-019-2078-7 (PMC6446277; doi:10.1186/s12888-019-2078-7)

Additional file 4 PRISMA flow chart

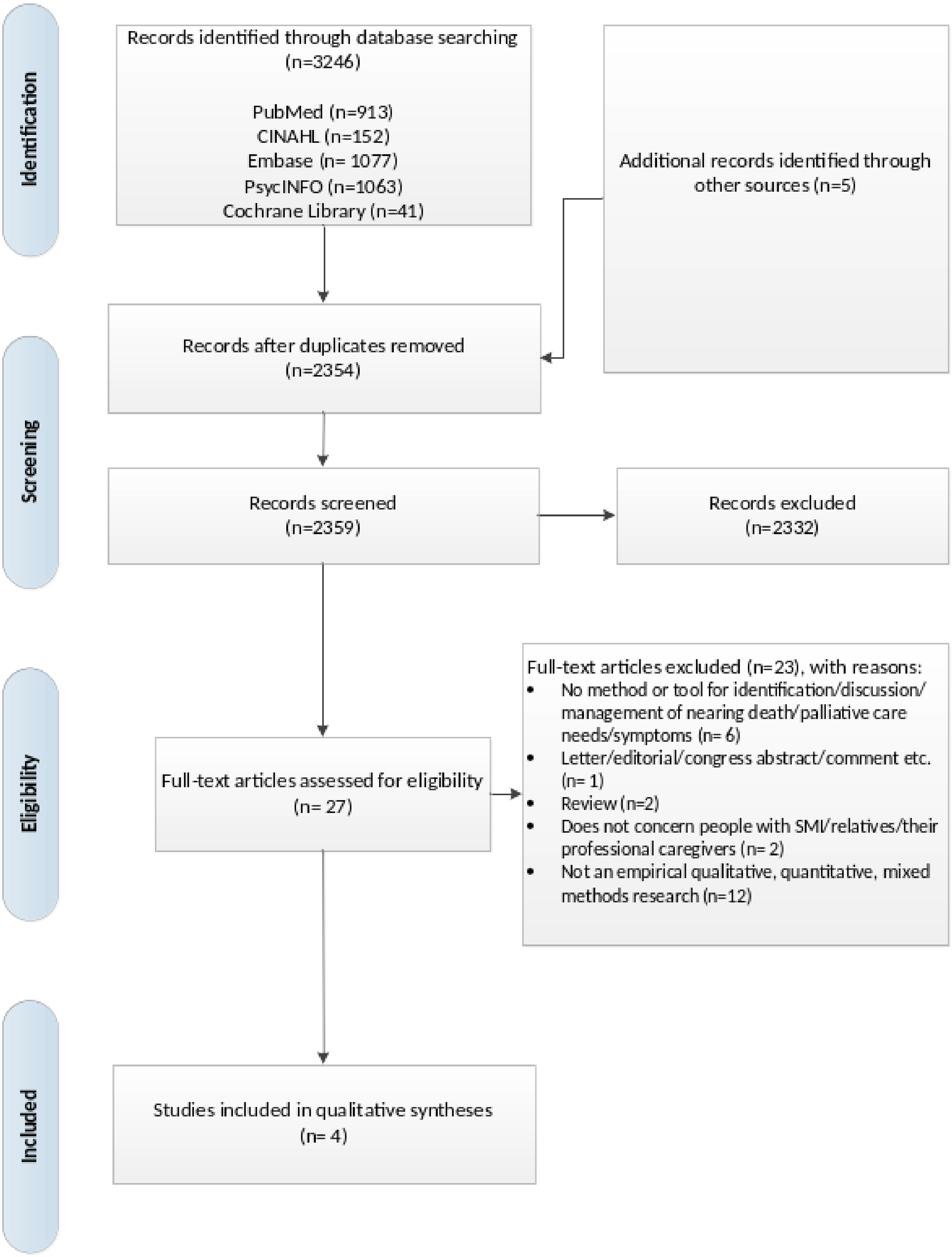

Supplement: Supplementary file 4 [file 12888_2019_2078_MOESM4_ESM.docx]
